# Supplementary material for: Effects of Organophosphate-Degrading Bacteria on the Plant Biomass, Active Medicinal Components, and Soil Phosphorus Levels of Paris polyphylla var. yunnanensis
Source: Plants (Basel). 2023 Jan 31;12(3):631. doi: 10.3390/plants12030631 (PMC9921132; doi:10.3390/plants12030631)
Supplement: Supplementary file 1 [file plants-12-00631-s001.zip › plants-2172742-supplementary.pdf]

**Table S1.** Linear equations, correlation coefficients, and linear ranges for steroidal saponins

| Name | Regression equation | $R^2$ | Linear range（μg·mL$^{-1}$） |
|---|---|---|---|
| Pseudoprotodioscin | Y=6927.7X-14494.0 | 0.9992 | 23.60–188.80 |
| Polyphyllin VII | Y=6202.0X-3225.4 | 0.9997 | 20.65–165.20 |
| Polyphyllin H | Y=6513.7X-9075.1 | 0.9992 | 23.25–186.00 |
| Polyphyllin II | Y=6141.8X-6846.1 | 0.9991 | 20.55–164.40 |
| Dioscin | Y=6703.0X-14192.0 | 0.9994 | 30.65–245.20 |
| Polyphyllin I | Y=6898.5X-30902.0 | 0.9996 | 31.85–254.80 |

**Table S2.** Steroidal saponin spiked recovery experiments

| Name | Weighing volume (g) | Saponin content (μg) | Amount added (μg) | Measured amount (μg) | Recovery rate (%) | Mean value (%) | RSD (%) |
|---|---|---|---|---|---|---|---|
| Pseudoprotodioscin | 0.2504 | 31.801 | 23.600 | 54.399 | 95.75 | 95.80 | 1.10 |
| | 0.2502 | 31.775 | 23.600 | 54.449 | 96.07 | | |
| | 0.2509 | 31.864 | 23.600 | 54.264 | 94.92 | | |
| | 0.2508 | 31.852 | 23.600 | 54.327 | 95.23 | | |
| | 0.2501 | 31.763 | 23.600 | 54.854 | 97.84 | | |
| | 0.2505 | 31.814 | 23.600 | 54.225 | 94.96 | | |
| Polyphyllin VII | 0.2504 | 246.143 | 206.500 | 450.188 | 98.81 | 98.40 | 1.79 |
| | 0.2502 | 245.947 | 206.500 | 449.714 | 98.68 | | |
| | 0.2509 | 246.635 | 206.500 | 445.664 | 96.38 | | |
| | 0.2508 | 246.536 | 206.500 | 445.937 | 96.56 | | |
| | 0.2501 | 245.848 | 206.500 | 449.687 | 98.71 | | |
| | 0.2505 | 246.242 | 206.500 | 455.381 | 101.28 | | |
| Polyphyllin H | 0.2504 | 113.431 | 116.250 | 231.918 | 101.92 | 101.75 | 2.76 |
| | 0.2502 | 113.341 | 116.250 | 234.144 | 103.92 | | |

| | | | | | | | |
|---|---|---|---|---|---|---|---|
| | 0.2509 | 113.658 | 116.250 | 228.261 | 98.58 | | |
| | 0.2508 | 113.612 | 116.250 | 230.523 | 100.57 | | |
| | 0.2501 | 113.295 | 116.250 | 236.397 | 105.89 | | |
| | 0.2505 | 113.477 | 116.250 | 229.248 | 99.59 | | |
| Polyphyllin II | 0.2504 | 696.112 | 616.500 | 1306.988 | 99.09 | 98.81 | 0.88 |
| | 0.2502 | 695.556 | 616.500 | 1309.100 | 99.52 | | |
| | 0.2509 | 697.502 | 616.500 | 1303.691 | 98.33 | | |
| | 0.2508 | 697.224 | 616.500 | 1309.325 | 99.29 | | |
| | 0.2501 | 695.278 | 616.500 | 1308.215 | 99.42 | | |
| | 0.2505 | 696.390 | 616.500 | 1295.831 | 97.23 | | |
| Dioscin | 0.2504 | 82.382 | 91.950 | 176.004 | 101.82 | 102.52 | 0.90 |
| | 0.2502 | 82.316 | 91.950 | 176.108 | 102.00 | | |
| | 0.2509 | 82.546 | 91.950 | 175.985 | 101.62 | | |
| | 0.2508 | 82.513 | 91.950 | 176.733 | 102.47 | | |
| | 0.2501 | 82.283 | 91.950 | 177.395 | 103.44 | | |
| | 0.2505 | 82.415 | 91.950 | 177.852 | 103.79 | | |
| Polyphyllin I | 0.2504 | 163.511 | 159.250 | 320.222 | 98.41 | 98.72 | 0.84 |
| | 0.2502 | 163.381 | 159.250 | 320.966 | 98.95 | | |
| | 0.2509 | 163.838 | 159.250 | 318.762 | 97.28 | | |
| | 0.2508 | 163.772 | 159.250 | 320.892 | 98.66 | | |
| | 0.2501 | 163.315 | 159.250 | 321.857 | 99.56 | | |
| | 0.2505 | 163.577 | 159.250 | 321.995 | 99.48 | | |
